# Structure—Function relationships of equine menisci

**DOI:** 10.1371/journal.pone.0194052

**Published:** 2018-03-09

**Authors:** Iris Ribitsch, Christian Peham, Nicole Ade, Julia Dürr, Stephan Handschuh, Johannes Peter Schramel, Claus Vogl, Heike Walles, Monika Egerbacher, Florien Jenner

**Affiliations:** 1 Department for Companion Animals and Horses, Veterm, University Equine Hospital, Vetmeduni Vienna, Vienna, Vienna, Austria; 2 Department of Health Sciences and Technology, Institute for Biomechanics, ETH Zurich, Zurich, Zurich, Switzerland; 3 Department of Pathobiology, Unit of Histology and Embryology, Vetmeduni Vienna, Vienna, Vienna, Austria; 4 Vetcore Facility for Research, Vetmeduni Vienna, Vienna, Vienna, Austria; 5 Department of Biomedical Sciences, Unit of Molecular Genetics, Vetmeduni Vienna, Vienna, Vienna, Austria; 6 Department of Tissue Engineering and Regenerative Medicine (TERM), University Hospital Wuerzburg and Translational Center Wuerzburg, Wuerzburg, Baveria, Germany; University of Zaragoza, SPAIN

## Abstract

Meniscal pathologies are among the most common injuries of the femorotibial joint in both human and equine patients. Pathological forces and ensuing injuries of the cranial horn of the equine medial meniscus are considered analogous to those observed in the human posterior medial horn. Biomechanical properties of human menisci are site- and depth- specific. However, the influence of equine meniscus topography and composition on its biomechanical properties is yet unknown. A better understanding of equine meniscus composition and biomechanics could advance not only veterinary therapies for meniscus degeneration or injuries, but also further substantiate the horse as suitable translational animal model for (human) meniscus tissue engineering. Therefore, the aim of this study was to investigate the composition and structure of the equine knee meniscus in a site- and age-specific manner and their relationship with potential site-specific biomechanical properties. The meniscus architecture was investigated histologically. Biomechanical testing included evaluation of the shore hardness (SH), stiffness and energy loss of the menisci. The SH was found to be subjected to both age and site-specific changes, with an overall higher SH of the tibial meniscus surface and increase in SH with age. Stiffness and energy loss showed neither site nor age related significant differences. The macroscopic and histologic similarities between equine and human menisci described in this study, support continued research in this field.

## Introduction

In accordance with the role menisci play in knee joint function, meniscal injuries are common in athletes and the general population [1]. The cumulative population risk of a meniscal injury requiring surgery between the ages of 10 and 64 years is estimated to be 15% with 50% of patients developing osteoarthritis (OA) within 10–20 years after injury [1]. Accordingly, the need to improve treatment for meniscal injuries and thus to identify appropriate translational animal models for meniscus tissue engineering and regenerative repair is of critical importance. The horse (*Equus caballus*), as one of the few species suffering from naturally occurring meniscus injuries and dysfunction, lends itself for this role as it would not only serve as an animal model but also as a beneficiary of improvements in the treatment. However, while comparable meniscal pathology can substantiate the validity of a species as translational animal model [2], the anatomical, physiological and biomechanical properties also need to approximate the conditions in humans. Although, the anatomy of equine menisci is well known [3, 4],, the histologic composition and biomechanical properties of the equine meniscus still need to be characterized, prior to using the horse as a translational model to study meniscus disorders.

Humans and quadrupeds have strikingly similar meniscal structures and share the relevant knee anatomy including cruciate ligaments, menisci, asymmetrical collateral ligaments and a bi-condylar distal femur [2–7], but small animals like rabbits or rats walk in a much more flexed knee position compared to humans or larger animals [8]. While no animal model, including primates [2] can completely emulate the knee hyperextension seen in humans, the gait of horses, sheep and goats is considered to most closely resemble the human [9]. The equine standing femorotibial joint angle of 150° approximates the almost 180° angle of humans. Also the cranio-caudal translocation of equine menisci during knee flexion and extension is similar to the human [10]. Furthermore, hyperextension can cause pathological forces and injuries in the cranial horn of the equine medial meniscus, analogous to those observed in the human posterior medial meniscal horn upon hyperflexion [11]. Meniscus injuries are thus also a common cause of lameness in horses and the most common soft tissue injury in the equine femorotibial joint [12, 13].

Analogous to the anatomy, the generic description of menisci being composed primarily of an interlacing network of collagen fibres (mainly collagen type I (Col I)), meniscal cells and extracellular matrix (ECM; water, collagen and proteoglycans (PG)) holds true regardless of species [2, 14]. The collagen fibres are arranged in three different layers and patterns: A thin superficial meshwork of fibrils followed by a lamellar layer with radially oriented fibrils and a central layer of circular fibre bundles interwoven with a few radial bundles [15]. In the transverse section three zones differing in vascularization and neural supply can be distinguished: the abaxial vascular/neural (red-red) zone, the middle mixed (red-white) zone and the axial avascular/aneural (white-white) zone [16–19]. Menisci behave like viscoelastic, anisotropic, biphasic structures with the interstitial water constituting the fluid phase and a porous-permeable solid phase composed of the collagen network and glycosaminoglycans (GAGs) [20]. Their complex natural architecture and anisotropic, biphasic composition allows for an optimal redirection and resistance of compressive and circumferential forces as well as shear and hoop stress [21]. Due to the regionally differing PG and collagen contents, as well as collagen fibre orientation, the mechanical properties of the meniscus, the stress–strain and fluid flow environment may vary greatly with location [22, 23]. In addition to the regional differences, meniscal structure and mechanical properties are influenced by species, activity level, age and degree of degeneration [2, 24–27]. However, it is not known yet which meniscus constituents at different depths and locations contribute to the meniscus’ biomechanical properties. For instance the meniscus’ superficial layer (SL) is considered to play an important role for its integrity, function and mechanical properties [28]. It is believed to govern compressive forces, provide a low friction surface to the contacting articular cartilage of the tibia and femur and to be pivotal for allowing fluid movement while maintaining basic function [28, 29]. Therefore, the aim of this study was to investigate the composition and structure (ECM, Col I fibre network and GAG content) of the equine knee meniscus in a site and age specific manner and to further elucidate their potential relationships with site-specific biomechanical properties.

We hypothesized that 1) due to differences in loading at different meniscal sites during the equine gait cycle, different stress concentrations may be distributed across the meniscus, potentially leading to site-specific structural and hence biomechanical properties; and that 2) the site specific mechanical properties may be subjected to age related changes.

Analysis of material properties at the nano-scale level coupled with other larger scale properties such as tensile, compressive or shear properties is considered to accurately model a meniscus for the ultimate goal of creating an effective meniscal replacement that can mimic the native human menisci [28]. Therefore we analysed and compared the shore hardness (SH) as well as stiffness (ST) and energy loss (EL) during loading and unloading among the three distinct anatomic regions (anterior horn (region A), pars intermedia (region B) and posterior horn (region C)) of the lateral and medial menisci of young, middle-aged and old horses, using a combination of nano-scale and compressive analysis approaches.

## Materials and methods

### Sample collection

All horses included in the study (n = 23) were euthanized for reasons unrelated to this study and had no signs of musculoskeletal disease related to the stifle joint. The animal owner’s written consent to collect and analyse the menisci and to publish resulting data was obtained according to the standard procedure of the University of Veterinary Medicine Vienna. All menisci were obtained within 12 hours post mortem, examined macroscopically and checked for potential injuries or damage they may have sustained upon harvesting. Age, sex and breed of all horses were documented. Donors were divided into young (0–4 years), middle-aged (5–16 years) and old (16–25 years) age groups.

Thirteen (13/23) paired medial and lateral menisci obtained from young (n = 2), middle-aged (n = 8) and old (n = 3) horses were used to establish the link between histology and biomechanics. For this purpose, one meniscus pair (from either the left or right leg) of each horse was randomly assigned to histologic evaluation and the contralateral to biomechanical testing. Paired menisci of one randomly selected middle-aged horse (9 years) were further subjected to microCT scanning.

To raise the power of the biomechanical analysis both (left and right) meniscus pairs of ten (10/23) horses (whose menisci did not undergo histologic evaluation) underwent biomechanical testing. The same quality criteria for inclusion into the study were applied: Horses free of musculoskeletal disease related to the stifle joint, menisci collected within 12 hours post mortem, no injuries or damage sustained upon harvesting. Hence in total, 33 meniscus pairs from 23 horses (young, n = 5; middle-aged, n = 8; old, n = 10) underwent biomechanical testing.

Samples assigned to biomechanical testing were wrapped in phosphate buffered saline (PBS, DPBS with Ca, Mg, Lonza) soaked gauze and frozen at -20° until mechanical testing. Samples for histology and microCT scanning were kept in 4% buffered formalin (ACM, Herba Chemosan Apotheker AG) until further processing.

### Structural evaluation

#### Histological analysis

**Fig 1:**
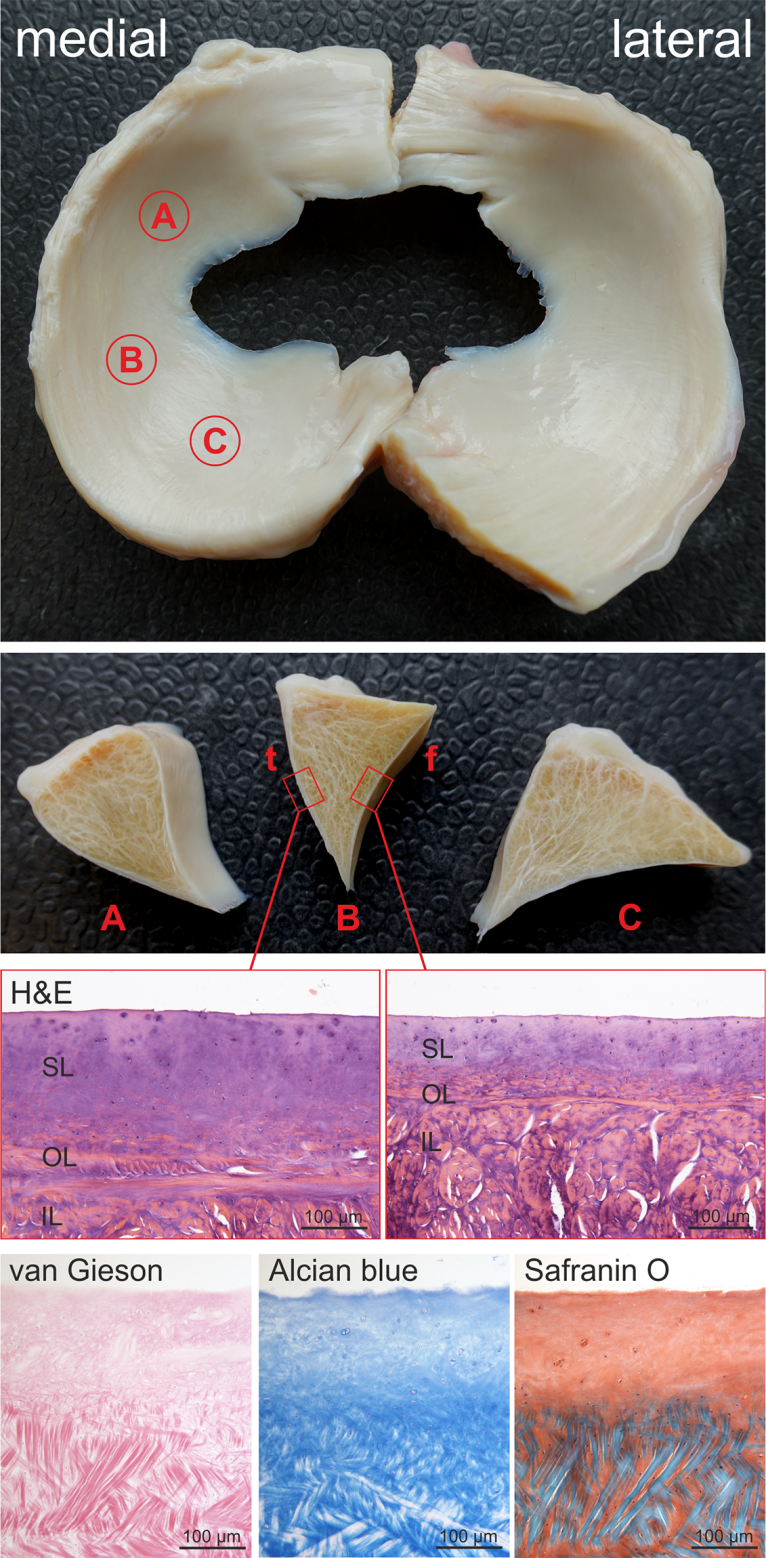
The equine meniscus. Top Row: Macroscopic picture of a lateral and medial meniscus of a horse indicating the different anatomic regions tested (A: anterior horn, B: pars intermedia, C: posterior horn). Second Row: Cross sections of each anatomic region (t: tibial, f: femoral). Third Row: Different thickness of the superficial layer at the tibial and femoral meniscus surface shown in histologic sections stained with H&E (SL = superficial layer, OL = outer layer, IL = inner layer). Bottom Row: Collagen fiber orientation (van Gieson) and GAG composition (Alcian blue and Safranin O) in the respective layers (region B, femoral side).

Of the thirteen paired medial and lateral menisci which underwent histologic evaluation, samples were taken from the three anatomic regions A, B and C (Fig 1) and processed as described previously [30].

Staining for Haematoxylin and Eosin, to illustrate fibre networks, as well as the thickness of the SL, Safranin O / Fast Green and Alcian blue, to show ECM composition and Van Gieson, for illustration of collagen fibre orientation, were performed as described previously [30].

#### MicroCT analysis

In addition to the histological evaluation, microCT scans were acquired from regions A, B, and C of one exemplary meniscus pair (medial and lateral) of a middle-aged horse (9 years). As a proof of principal, we tested the potential use of micoCT imaging for the quantitative structural evaluation of the meniscus. The scans supplement the histological data by providing an overview of the size and shape of the meniscus and by illustrating relevant topographic characteristics.

Compared to traditional histology, microCT analysis offers two crucial benefits. First, microCT facilitates more accurate measurement of layer thickness, as virtual sections can be precisely aligned in the image volume and parameters can be measured on consecutive virtual slices in a given sub-volume (like in the present study: 30 slices covering roughly 2mm). Second, microCT images are accurate geometric representations of the object, while histological sectioning potentially introduces geometric distortions that can strongly affect area measurements.

Cross-sections of 2 mm from regions A, B and C were obtained. Samples were fixed in 4% formalin and stained with 1% (w/v) phosphotungstic acid (PTA) in 70% ethanol for 21 days. PTA has a strong affinity for binding to collagen [31]and has been used before as a contrast agent for microCT imaging [32, 33]. After staining, samples were imaged using a Scanco μCt 35 (SCANCO Medical AG, Brüttisellen, CH) at 18.5μm isotropic voxel size.

MicroCT scans were evaluated using Amira 6.2 (FEI Visualization Sciences Group, Mérignac Cédex, France). For each region, 30 cross-sectional virtual slices were analysed by first segmenting the total cross-sectional area of the meniscus and subsequently segmenting the SL using a uniform threshold on the standardized image intensities. Finally, the SL was divided into three sub-sections: SL of the femoral meniscus surface, SL of the tibial meniscus surface, and axial tip of the SL which could not be assigned either to the femoral or tibial surface. Based on this segmentation the following parameters were calculated for each sample to give an exemplary idea of the sizes and proportions: average cross-sectional area of the total meniscus, average cross-sectional area of the SL, average cross-sectional area of the axial tip of the SL, average thickness of the SL at the femoral surface, and average thickness of the SL at the tibial surface.

### Biomechanical testing

#### Shore hardness

Prior to mechanical testing the menisci were thawed at room temperature for 24 hours. The SH was determined by indentation technique at the tibial and femoral surface (region A, B and C) using a PCE-DD-A Shore A durometer (PCE Instruments, Germany) with a resolution of 0.5 and a sensitivity of +/- 2 hardness grades. The penetration depth (PD) of a particular SH is calculated according to the following formula *PD* = 2.5 − *SH* * 0.025, at a vertical load of 12.5N and measurement range between 0 and 100 Shore A. This results in a PD of 2.5mm at SH = 0 and a PD of 0mm at SH = 100. The indenter had a truncated conical tip with a cone angle of 35 ± 0.25°and an end plane diameter of 0.79 ± 0.01 mm, chosen to be small enough to minimize effects from the sample edges [34, 35].

#### Stiffness and energy loss

**Fig 2:**
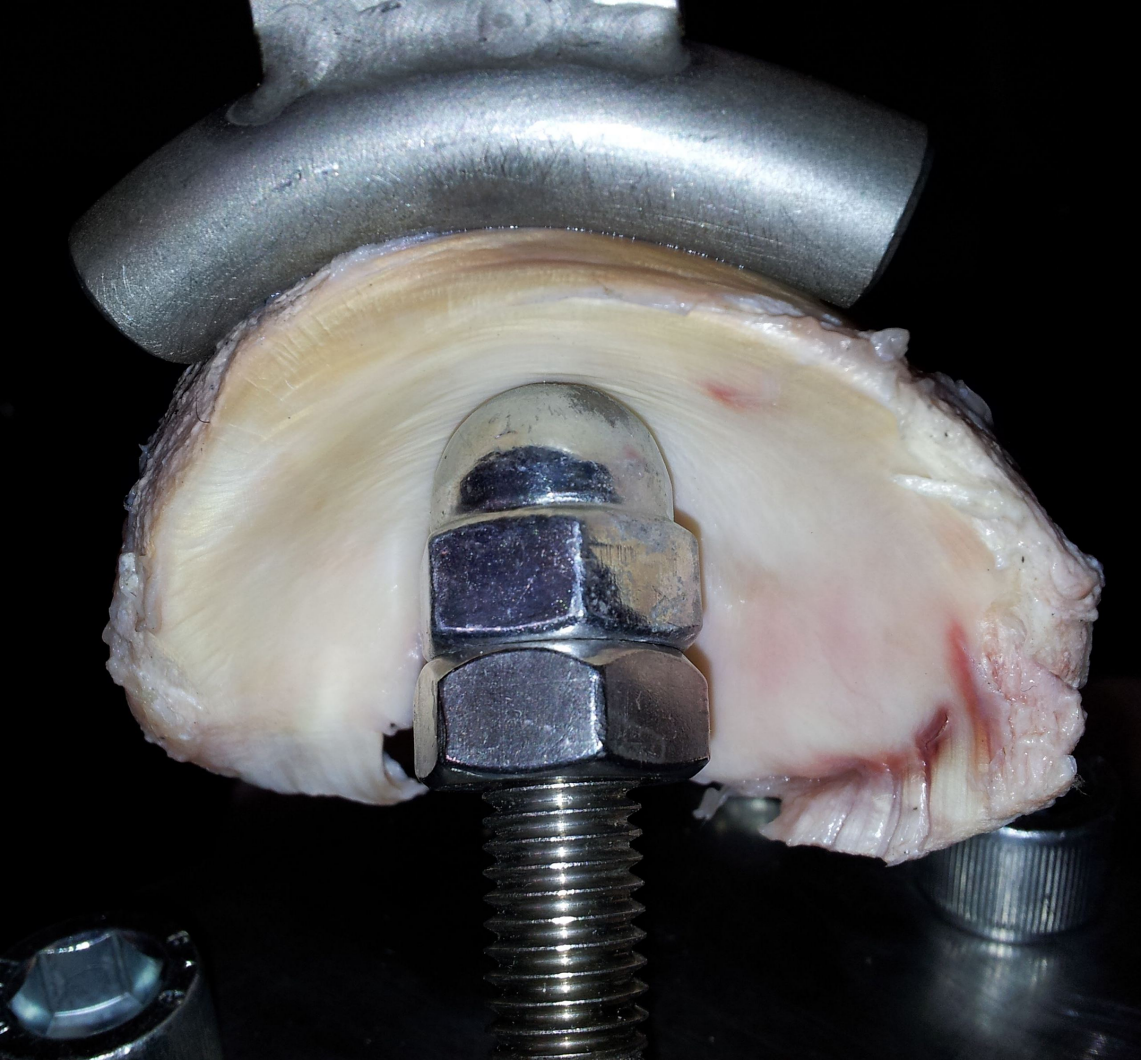
Biomechanical testing device for determination of stiffness and energy loss. Equine Meniscus mounted onto a custom made, curved jig to apply uniaxial compressive forces for determination of stiffness and energy loss.

**Fig 3:**
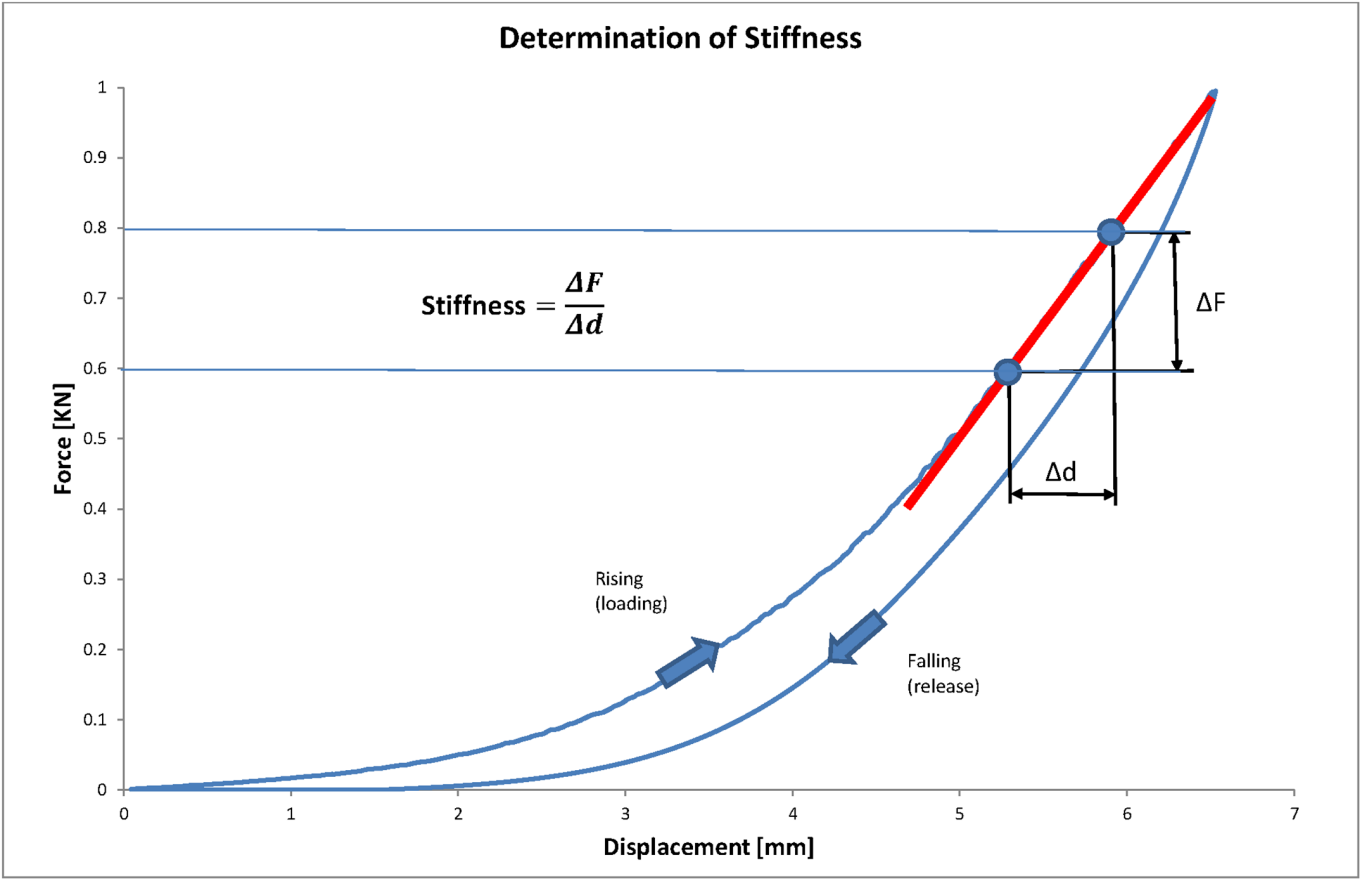
Meniscus´ stiffness. Meniscus´ stiffness was calculated as the slope of the load-deformation curve.

**Fig 4:**
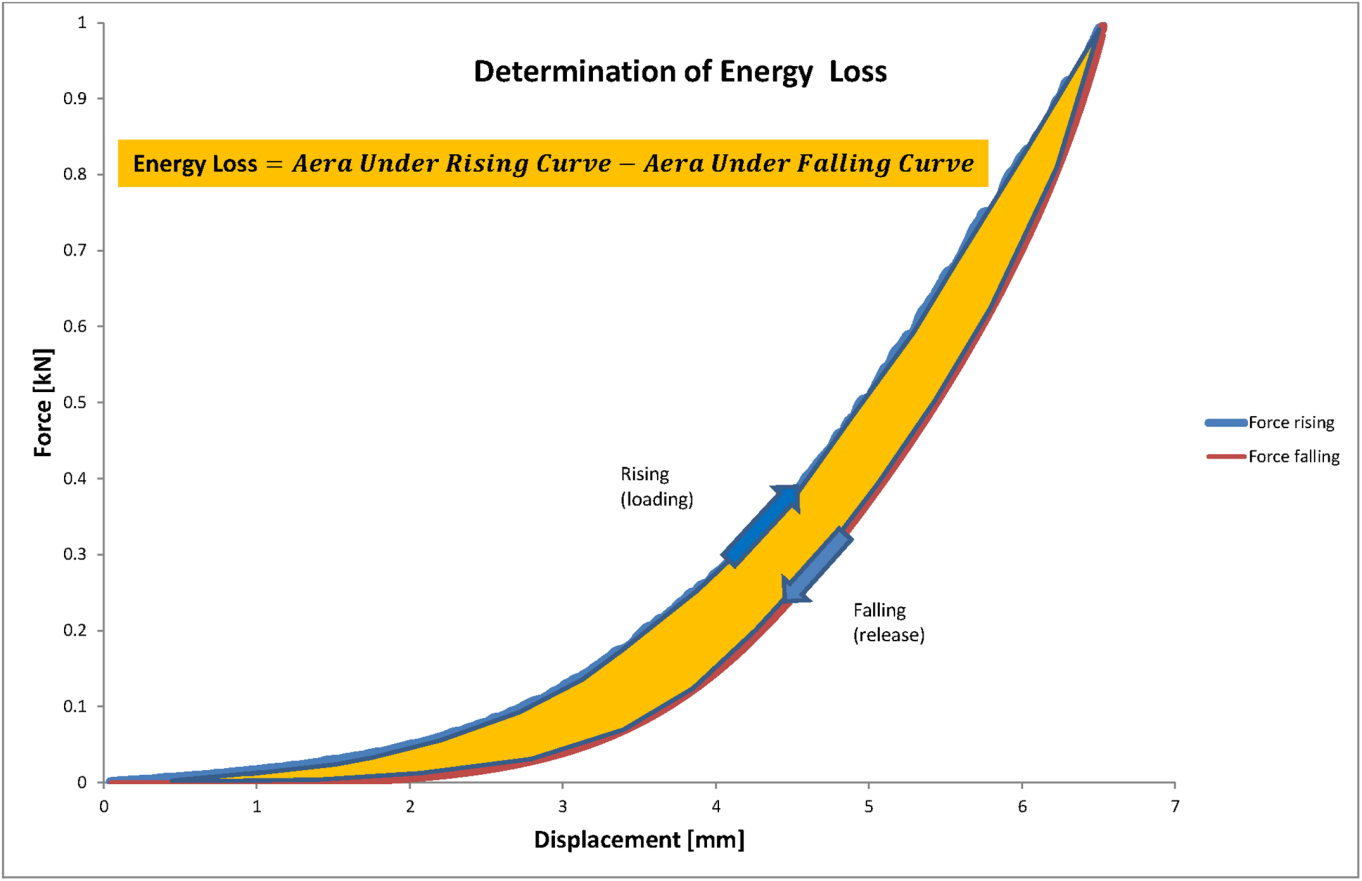
Meniscus´ energy loss. Meniscus´ energy loss was calculated using the integral of the stress–strain curve during loading.

Uniaxial compressive testing was used to determine the ST and EL at the three anatomic regions of each meniscus. Menisci were mounted onto a custom made curved jig with a radius of 32mm (Fig 2), which allowed testing the menisci with a distribution of force in a physiologic manner independent of different meniscal sizes and shapes. The spherical shape of the actuator tip (diameter 15.8 mm) was designed to ensure a consistent contact area with the concave contour of the femoral meniscal surface but to avoid shear stress and stress concentration at the contact area. The three anatomic regions of each meniscus were tested separately in random sequence using a Walter+Bai AG material testing system. Each sample was compressed at a constant displacement rate of 0.5 mm/s to a maximum load of 1000 N (1kN) and subsequently unloaded. Applied load (N) and displacement (mm) were recorded. Specimens that slipped during the test were discarded from analysis. Stiffness was calculated as the slope of the loading curve ($Stiffness\;=\;\frac{\Delta F}{\Delta d}$, see Fig 3). Energy loss was calculated using the integral of the load–deformation curve during loading/unloading (*Energy loss* = *area under loading curve* − *area under releasing curve*, see Fig 4).

### Statistical analysis

Target variables were not transformed as no obvious deviations from linear model assumptions were found. If data from left and right menisci were available for a horse, the horse’s ID was entered as a factor to avoid pseudo-replication. Regressions with age were calculated for single variables (femoral SH, tibial SH, stiffness, energy loss) in different regions (lateral vs. medial, anatomical regions) of the menisci. Furthermore, for each of the four variables, ANCOVAs with age as regression variable and the different regions as factor were performed. Correlations between corresponding variables on left and right legs were calculated. Tests of correlations among variables, e.g., femoral and tibial SH, or SH and stiffness, or SH and energy loss, were also performed. For some tests, averages among regions (e.g.: lateral and medial) were calculated, in addition to separate analyses for each of the four regions.

### Comparison of structural and biomechanical properties

All results from histology and biomechanical testing (SH, ST, EL) were compared between the three different age groups (young, middle-aged and old), the three anatomic regions (A, B and C) and medial versus lateral menisci. Histology results as well as SH were additionally compared between the tibial and femoral meniscus surface.

## Results

### Structural evaluation

#### Histology

The histologic architecture, the fibre types and orientation, the size and shape of the fibrochondrocytes, as well as differences in vascularization between the three zones and layers we described in detail in [30]. In the current paper we focused on the two major matrix components (Col I fibres and GAGs) and the question whether differences in their arrangement, network formation and distribution at regions A, B and C, between medial and lateral menisci and between the three age groups may account for differences in biomechanical properties.

On the basis of collagen morphology and arrangement, three distinct meniscal layers can be discriminated: The SL, which mantles the meniscus at the femoral as well as tibial surface, and two deep layers—an outer (OL) and inner (IL) layer (Fig 1). Clear differences were detected between the thickness of the SL when comparing the tibial and femoral side of the meniscus, with the tibial side being significantly thicker than the femoral side in all regions except for region C (Fig 1). Collagen fibres in the SL, which was characterized by a meshwork of very thin fibres, and the OL showed no distinct fibre orientation. In the IL, which accounts for the main portion of the equine meniscus, the mainly thick collagen fibres were circumferentially oriented with a strictly parallel alignment in the red-red zone interrupted only by a few radially oriented branches of connective tissue. The red-white and white-white zone, were additionally interwoven with collagen fibre bundles oriented in a proximo-distal direction. No age related changes or differences between regions A, B and C were detected.

**Fig 5:**
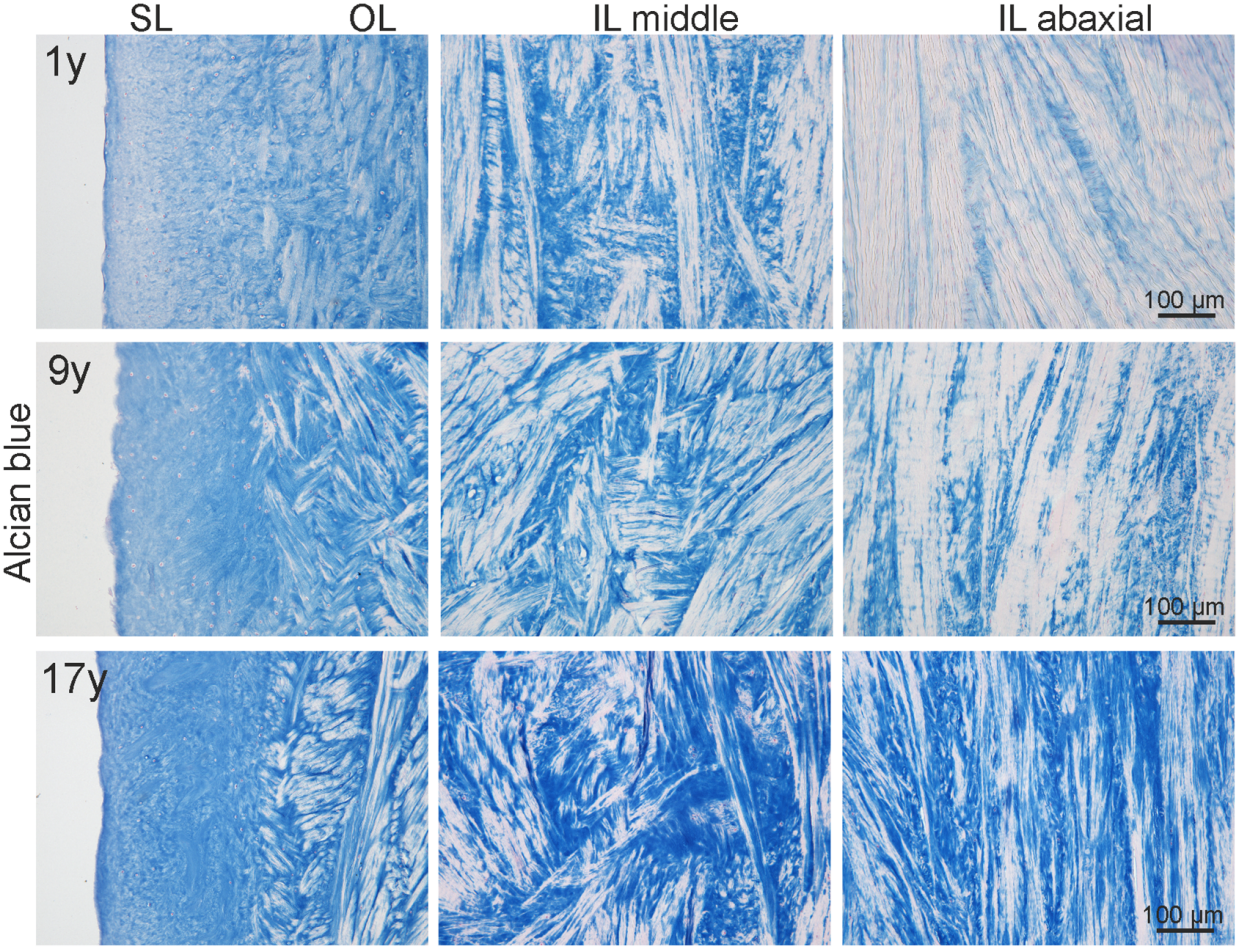
Age and topographic differences in GAG content. Representative micrographs showing age related increase of GAG production (Alcian blue staining) in the SL, OL and IL (middle and abaxial zone) of a 1 year (y), 9y and 17y old horse (all pictures from lateral menisci, region B). Scale bars as depicted.

The GAG content and distribution was subject to age dependent as well as topographic differences (Fig 5). In young horses, the two axial zones of the meniscus contained more GAG than the abaxial zone, which seemed to be almost free of GAGs despite single and small areas surrounding some fibrochondrocytes. The abaxial zone of middle-aged and old horses in contrast stained clearly positive for GAGs. The two axial zones were positive for GAGs among all age groups particularly adjacent to fibrochondrocytes. In young menisci, GAGs were generally more evenly distributed whereas menisci of older horses showed distinct positive or negative areas. The collagen fibrils of the SL were masked by GAG in horses of all age groups. In addition, GAG staining seemed to be overall less marked in the medial menisci compared to the lateral. However, between regions A, B and C no differences were detected.

#### MicroCT analysis

MicroCT proved to be a valuable tool for accurately measuring both cross-sectional area and thickness of the superficial layer in one exemplary pair of menisci.

We demonstrate that PTA provides sufficient contrast to distinguish the SL from deeper layers, and that intensity-based segmentation allows to quantify the thickness of the SL. While this is an encouraging finding, the present study does not aim to make a strong quantitative statement on absolute SL parameters, as this would require analysing a larger number of specimens.

**Fig 6:**
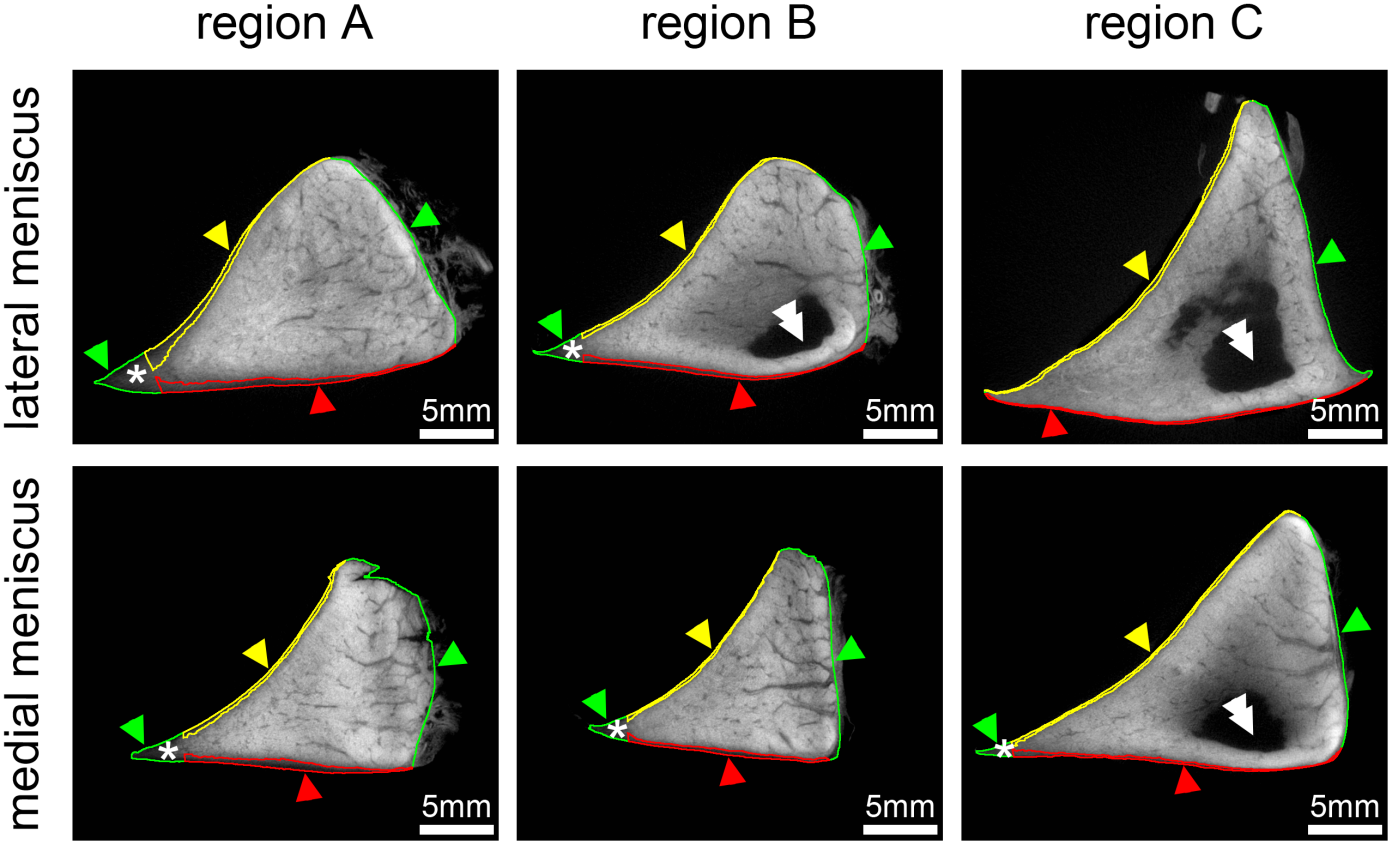
MicroCT analysis of the equine meniscus. Meniscus samples were stained with phosphotungstic acid (PTA). The PTA stain allowed discriminating the superficial layer (SL) from the outer and inner deep layers of the meniscus based on lower staining intensity. Colour contours show the result of segmentation. Cross sectional area was largest for region C in the lateral and medial meniscus, while the thickness of the SL at both, the femoral and tibial surface, as well as the area of the axial tip of the SL was largest in region A for the lateral and medial meniscus. Yellow arrowheads and contour = SL at femoral surface; red arrowheads and contour = SL at tibial surface; green arrowheads and contour = outer meniscus contour; asterisk = axial tip of SL, double arrowheads = unstained regions of samples due to limited tissue penetration properties of PTA.

MicroCT analysis confirmed common anatomic knowledge [3, 36, 37] that cross-sectional area is largest in the posterior horn (region C) followed by the anterior horn (region A), and smallest in the pars intermedia (region B). All measurements are summarized in S1 Table, and representative cross-sections for each analysed region are shown in Fig 6. For both menisci the area of the SL was largest in region A. Also the area of the axial tip of the SL was largest in region A. As also seen in the histologic sections (Fig 1), the thickness of the SL was higher at the tibial surface compared to the femoral surface for all regions except for region C in the lateral meniscus. The thickness of the SL was largest in region A both for the femoral surface and the tibial surface.

### Biomechanical testing

#### Shore hardness, stiffness and energy loss

The biomechanical test results of the equine lateral and medial menisci at regions A, B and C are detailed in Tables 1 and 2. Overall the SH of equine menisci was found to be similar to car tires [38]. The energy loss was highest in region A and lowest in region B both laterally and medially, with the medial meniscus showing higher energy losses and greater stiffness. However, the differences did not reach statistical significance.

**Table 1:** Equine meniscus´ biomechanical properties.

		lat—A	lat—B	lat—C	med—A	med—B	med—C
**Shore hardness tibial [Shore A]**	**mean**	58.40	60.94	65.20	60.33	58.98	65.33
	**sd**	7.11	7.06	7.82	6.15	8.53	6.96
	**min**	48.00	47.75	50.25	52.25	44.25	52.5
	**max**	68.75	72.00	85.00	72.5	75.75	77.00
**Shore hardness femoral [Shore A]**	**mean**	50.85	42.73	42.68	44.2	42.73	46.00
	**sd**	7.94	5.09	7.92	7.6	6.18	7.21
	**min**	41.00	33.5	32.00	34.75	32.35	34.00
	**max**	63.75	48.5	59.00	57.5	51.00	56.00
**stiffness [N/mm]**	**mean**	0.434	0.499	0.492	0.498	0.515	0.443
	**sd**	0.086	0.094	0.065	0.312	0.132	0.109
	**min**	0.37	0.33	0.37	0.3	0.28	0.32
	**max**	0.59	0.64	0.59	1.12	0.75	0.74
**Energy loss [J]**	**mean**	1.16	0.637	0.67	1.256	0.83	1.00
	**sd**	0.415	0.345	0.411	0.481	0.475	0.526
	**min**	0.59	0.26	0.25	0.36	0.32	0.35
	**max**	1.78	1.49	2.09	1.76	1.93	1.92

Overview of results from biomechanical testing at different anatomic regions (A, B, C). Lat = lateral, med = medial, sd = standard deviation, min = Minimum, max = Maximum.

**Table 2:** Age related changes of the equine meniscus´ biomechanical properties.

		Shore hardness tibial [Shore A]		Shore hardness femoral [Shore A]		stiffness [N/mm]		Energy loss [Nm]	
		mean	sd	mean	sd	mean	sd	mean	sd
**lat—A**	**young**	51.750	4.023	48.300	4.065	0.401	0.038	1.588	0.272
	**middle**	62.120	6.541	58.000	4.243	0.374	0.000	0.880	0.000
	**old**	60.900	6.864	49.500	9.816	0.475	0.111	0.966	0.340
**lat—B**	**young**	53.350	6.810	40.166	4.500	0.583	0.064	0.790	0.475
	**middle**	62.090	6.260	46.375	3.000	0.460	0.080	0.537	0.179
	**old**	63.850	5.220	42.800	5.870	0.480	0.099	0.633	0.400
**lat—C**	**young**	59.200	6.066	39.660	8.620	0.495	0.020	0.680	0.251
	**middle**	67.780	5.600	42.625	0.530	0.480	0.093	0.550	0.190
	**old**	66.125	9.040	44.500	9.630	0.500	0.062	0.765	0.600
**med—A**	**young**	54.250	2.000	40.750	5.300	0.420	0.077	1.610	0.208
	**middle**	64.250	11.67	45.875	1.600	0.330	0.000	1.150	0.000
	**old**	62.400	2.880	45.600	10.100	0.604	0.450	1.053	0.600
**med—B**	**young**	49.600	4.990	37.000	5.020	0.610	0.110	0.900	0.370
	**middle**	61.625	7.300	47.250	5.300	0.466	0.080	0.772	0.500
	**old**	61.550	7.900	44.350	5.320	0.500	0.155	0.820	0.550
**med—C**	**young**	56.100	2.400	38.500	3.900	0.430	0.053	1.011	0.505
	**middle**	67.560	7.000	51.620	4.420	0.417	0.080	0.760	0.377
	**old**	68.150	4.130	48.250	6.330	0.480	0.160	1.470	0.640

Overview of age related changes of biomechanical properties tested at different anatomic regions (A, B, C). Lat = lateral, med = medial, sd = standard deviation, min = Minimum, max = Maximum, young = 0–4 years, middle = 5–16 years, old = 16–25 years.

### Statistical analysis

Initially, we performed regressions for the four variables (femoral and tibial SH, stiffness and energy loss) and regions (medial vs. lateral, anatomic regions A, B and C) separately. Age was shown to have a marginally significant influence on the tibial meniscal SH at the medial (p = 0.051) and lateral (p = 0.09) region B and medial region C (p = 0.054). There was no significant influence of age on femoral meniscal SH. Similarly, age showed also no influence on stiffness and energy loss.

We next performed ANCOVAs with age as regression variable and region as factor. For tibial SH, the influence of age was significantly positive (p = 0.01) as was region (p = 0.0002); in particular, position C was harder both medially and laterally than position B (p< 0.01). For femoral SH and energy loss, neither the influence of age nor of position was significant. For stiffness, the influence of age was insignificant and the overall influence of region was marginally significant (p = 0.096) and reached statistical significance only for medial position B in pairwise comparison with medial position C (p = 0.012).

Correlations of the four variables and regions between left and right sides were generally not significant at any position, with the exception of tibial SH at position B (rho = 0.808; p = 0.005) and femoral SH at position C (rho = 0.657; p = 0.04). Average tibial and femoral SH were correlated (rho = 0.677, p = 0.031). Tibial SH was significantly higher than femoral SH (p < 0.001). Separate comparisons of the respective regions gave qualitatively similar results as the average. Only in one case (tibial SH for region B), we found a significant correlation between medial and lateral SH (p = 0.041). No region had significant differences between mean tibial and femoral hardness.

## Discussion

Studying the biomechanical and compositional properties of equine menisci at various ages can broaden our understanding of pathophysiological processes in the aging meniscus, which may lead to altered meniscus function, injury and consequently secondary OA. However, this knowledge is not only important for the equine veterinary field, but will contribute to a thorough validation of the horse as translational model for human meniscus disorders. Hence, in this study we investigated the site-specific biomechanical properties, composition and structure of the equine knee meniscus with special focus on the Col I fibre network and GAG content. Our aim was to elucidate a potential relationship between site- and age-specific compositional and biomechanical properties.

Both menisci (lateral and medial) are highest and widest at the posterior horn followed by the anterior horn and smallest, respectively least wide, in the pars intermedia [3, 36, 37] (Fig 1). The results of the biomechanical properties determined at these regions did not reliably mirror these anatomic characteristics.

The histologic architecture of the menisci was found to differ between the axial, middle and abaxial zone as well as between the SL, OL and IL.

The equine SL, analogous to human SL, is composed of a tight meshwork of randomly oriented Col I fibres similar to that of the articular cartilage tangential fibre layer (Fig 1) [15, 29, 39]. We measured equine SL thickness of 140–370 μm in one pair of formalin-fixed menisci using microCT images (Fig 6 and S1 Table), which may of course be subject to inter-individual differences but was similar to the reported thickness of human SL (150–200 μm) [40]. However, the findings encourage future quantitative microCT studies on meniscus morphology.

The equine SL at the tibial surface was markedly thicker than at the femoral surface. This was not only observed macroscopically, but confirmed by histology and illustrated by microCT (Figs 1 and 6). The greater thickness may be partially responsible for the significantly higher shore hardness detected at the tibial compared to the femoral surface. However, as the penetration depth (at SH = 60 the PD is 1mm) was greater than the thickness of the SL, the SH represents composite properties of the SL and the underlying OL and hence cannot exclusively be attributed to the different SL thicknesses. An anatomic reason for the differences in SH may be the more normal compressive forces encountered at the tibial surface, which is nearly flat and providing a consistent contact area with the equally flat tibial plateau, as compared to the concave femoral surface [4]. We also detected significant differences between the tibial SH of C and B, which may be a consequence of loading differences during the gait cycle as suggested by Mononen et al [41]. In human menisci nano-indentation testing of the SL showed no significant differences among different regions or surfaces possibly owed to the homogeneity of the meniscal SL [15, 28, 40, 42]. Reasons for this discrepancy may either be due to species-specific differences or the different characteristics of the test method used in the current study. Also, the samples used in the human study were from people between 50 and 77 years of age, which would roughly correspond to the old group (16–25 years) tested in this study. Maybe a broader age distribution of the samples included into the human study would have led to more comparable results. Further investigation of the composition and mechanical properties of the SL may lead to a better understanding of the structure-function relationship at this crucial meniscus layer.

Increasing age correlated with rising SH but not stiffness or energy loss particularly in regions B and C at the tibial surface. Increasing shore hardness with age may lead to changes in the meniscal function, which potentially translate into excessive loads transmitted to the contacting articular cartilage surface.

In contrast to SH, stiffness and energy loss did not vary significantly among the anatomical regions. This corresponds to the homogeneity of the collagen fibre architecture at the different regions found in histology. Other constituents than the collagen fibre network, such as GAGs and water content, are not well characterized, but may have direct correlation with mechanical strength and viscosity in compression [43, 44]. However, also the small differences in GAG content and distribution between young (more even GAG distribution) and old (Fig 5), as well as medial (less marked staining for GAG) and lateral menisci found in this study did not lead to a significant impact on stiffness and energy loss.

Previous studies reported site-specific variation in the mechanical properties for excised meniscal samples [34, 45–47], which we could not confirm in this study. This may be due to differences in sample processing. In contrast to testing meniscal pieces, which would have compromised the intra-meniscal fluid pressure environment and tissue integrity, i.e. by loosening the collagen fibril tension, we chose to test whole menisci. Compression based studies performed in humans, focusing on the medial meniscus, have shown the meniscus to be weakest in the posterior region [24, 26, 48], possibly elucidating why the majority of meniscal tears occur in the posterior meniscus horn [11]. For the horse in which the most common meniscal tears are isolated lesions of the cranial horn of the medial meniscus and its associated meniscotibial ligament [12, 49] no such relation could be found in this study. Maybe integration of a higher number of samples would have led to significance of potential minimal differences. Also investigation of potential differences of the biomechanical properties of the three different zones (axial to abaxial) which are consistent with differences in collagen composition, collagen fibre architecture, GAG content and vascularisation [30] rather than regions (A, B and C) could be productive to reveal biomechanical site-specific differences.

In summary our results suggest that the superficial meniscus layer may contribute to the meniscus SH and may play a pivotal role for meniscus function as is suspected for human menisci. The equine SL also has similar histologic architecture as described for human menisci. The SH of equine menisci is subjected to age as well as site-specific changes, with an overall higher SH of the tibial surface and increase in SH with age. For the gross mechanical testing of the whole menisci, no significant differences, neither site nor age related, could be shown with regard to ST and EL. Future studies, focusing on local biomechanical differences of the SL, OL and IL and at the different zones (axial, middle and abaxial), could further contribute to elucidating the correlation of meniscus biomechanical properties with durability, resilience, resistance to strain, shock absorption, or predisposition to degeneration. The macroscopic and histologic parallels between human and equine menisci established in this study and our previous study [30], support continued research in this field.

## Supporting information

S1 Table MicroCT results. Overview of site specific differences in cross sectional area size and thickness of the meniscus’ superficial layer (SL) as determined by microCT analysis of the different anatomic regions (A, B, C,) of one exemplary lateral and medial meniscus. For each region, 30 consecutive slices (slice thickness = 18.5μm) were analysed and averaged. lat = lateral, med = medial.
(DOCX)
