# Supplementary material for: Structure—Function relationships of equine menisci
Source: PLoS One. 2018 Mar 9;13(3):e0194052. doi: 10.1371/journal.pone.0194052 (PMC5844599; doi:10.1371/journal.pone.0194052)
Supplement: S1 Table — Overview of site specific differences in cross sectional area size and thickness of the meniscus’ superficial layer (SL) as determined by microCT analysis of the different anatomic regions (A, B, C,) of one exemplary lateral and medial meniscus. For each region, 30 consecutive slices (slice thickness = 18.5μm) were analysed and averaged. lat = lateral, med = medial. (DOCX) [file pone.0194052.s001.docx]

**S1 Table. MicroCT results.**

|  | **average area** | **average area** | **average area** | **average SL thickness** | **average SL thickness** |
| --- | --- | --- | --- | --- | --- |
|  | **total meniscus (mm^2^)** | **SL (mm2)** | **SL axial tip (mm2)** | **femoral**  **surface**  **(mm)** | **tibial**  **surface**  **(mm)** |
| **lat- A** | 211.94 | 20.43 | 6.46 | 0.29 | 0.35 |
| **lat-B** | 184.34 | 10.97 | 2.35 | 0.16 | 0.25 |
| **lat-C** | 234.78 | 9.69 | 0.00 | 0.20 | 0.14 |
| **med-A** | 153.13 | 13.76 | 3.72 | 0.22 | 0.37 |
| **med-B** | 123.49 | 8.62 | 2.40 | 0.14 | 0.27 |
| **med-C** | 211.45 | 10.33 | 1.40 | 0.17 | 0.19 |
